# Chemical chaperon 4-phenylbutric acid improves cardiac function following isoproterenol-induced myocardial infarction in rats

**DOI:** 10.22038/IJBMS.2023.68183.14891

**Published:** 2023-03

**Affiliations:** 1 Pharmacology and Toxicology Department, Faculty of Pharmacy, Tabriz University of Medical Sciences, Tabriz, Iran; 2 Student Research Committee, Tabriz University of Medical Sciences, Tabriz, Iran

**Keywords:** 4-Phenylbutric acid, Autophagy, Chaperon, Isoproterenol, Myocardial infarction

## Abstract

**Objective(s):**

4-Phenyl butyric acid (4-PBA) is a chaperone-mediated autophagy (CMA) inducer, which eliminates unnecessary and damaged cellular components through lysosomal enzymes. It could reduce misfolded and unfolded proteins produced after myocardial infarction (MI) and can improve cardiac function. We aimed to investigate the effect of 4-PBA on isoproterenol-induced MI in rats.

**Materials and Methods:**

Isoproterenol (100 mg/kg) was injected subcutaneously for two consecutive days simultaneous with an intraperitoneal (IP) injection of 4-PBA at 20, 40, or 80 mg/kg at 24-hr intervals for five days. On day 6, hemodynamic parameters, histopathological changes, peripheral neutrophil count, and total anti-oxidant capacity (TAC) were evaluated. The expression of autophagy proteins was measured by using western blotting. 4-PBA significantly improved post-MI changes in hemodynamic parameters.

**Results:**

Histological improvement was found in 4-PBA 40 mg/kg (*P*<0.05). The neutrophil count in the peripheral blood significantly decreased in the treatment groups compared with isoproterenol. Furthermore, 4-PBA at 80 mg/kg significantly increased the serum TAC compared with isoproterenol (*P*<0.001). Western blotting showed a significant decrease in the P62 level (*P*<0.05) of 40 and 80 mg/kg 4-PBA treated groups.

**Conclusion:**

This study demonstrated that 4-PBA could have a cardio-protective effect against isoproterenol-induced MI, which can be due to autophagy modulation and oxidative stress inhibition. Obtaining effective results in different doses shows the need for an optimum degree of cell autophagic activity.

## Introduction

Myocardial ischemia is among the most important causes of morbidity and mortality worldwide, with a 42% increased prevalence in recent years (1). Myocardial ischemia occurs due to obstruction of coronary arteries and consequently decreased blood flow to the coronary arteries, leading to myocardial infarction (MI). Tissue remodeling, necrosis, fibrosis, hypertrophy, inflammation, and accumulation of neutrophils at the site of infarction and subsequently increased destruction due to the activity of proteolytic enzymes are among the consequences of MI (2). 

Physiological stresses such as oxidative stress occur in MI, causing several changes in cells, and affecting the structure and function of proteins. In this process, unfolded and misfolded proteins are also synthesized, which can cause further cardiac damage and apoptosis of cardiac cells. These proteins activate the unfolded protein response pathway, which can cause further tissue damage (3, 4). Autophagy is an intracellular pathway that helps to eliminate the unfolded and misfolded proteins and plays a critical catabolic role in cell survival against different stress types such as endoplasmic reticulum stress (3, 5). The process of autophagy can activate programmed cell death under specific circumstances and serves as an alternative to the apoptotic pathway (6). Autophagy is divided into three main types macro-autophagy, micro-autophagy, and chaperone-mediated autophagy (CMA) (7). Microautophagy is mediated by lysosomal action through direct engulfment of the cytoplasmic cargo. In macro-autophagy, an autophagosome with a bilayer membrane is formed and engulfs the damaged organelles and the unusable proteins. It then delivers them to a vacuole to destroy and discard them (8-10). CMA is a selective form of autophagy in mammals. In this process, some specific proteins containing the KFERQ amino-acid sequence are detected by the chaperones and unfolded. Subsequently, they are delivered into the lysosomes by lysosome-associated membrane protein type 2A and degraded (11). Macroautophagy and CMA, are both maximally switched on in response to stress. As the sequential activation of these two pathways, cross-talk between these stress-related autophagic pathways is investigated in different studies (12).

The effective proteins in the macro-autophagy pathway include Beclin1, P62, and LC3. By measuring the level of these proteins, the autophagic activity of the cells can be quantified (13). Evidence shows that Beclin1 regulates autophagy and membrane trafficking involved in physiological and pathological processes (14). P62 identifies the intracellular residues and takes the role of their elimination. Impairment of autophagy would lead to accumulation of P62 in cells, leading to cellular stress and eventual disease (15, 16). Thus, an increased level of this protein indicates decreased autophagy (17, 18). It was reported that blockage of CMA activates macroautophagy in cultured cells (19). Furthermore, a bidirectional cross-talk between macroautophagy and CMA was established which means changes in the activity of one of these pathways will affect the protein breakdown contribution of the other pathway (20).

In the process of autophagy, the cytoplasmic form of LC3 (LC3-I) is converted to conjugated LC3 (LC3-II), which is absorbed by the autophagosomal membrane. Since LC3-II within the autophagosomal membrane is degraded by the lysosomal hydrolase, the transient amount of LC3-II cannot indicate autophagic activity. Thus, LC3-II lysosomal turnover is estimated to determine autophagic activity (21, 22). The GAPDH protein is used as a standard along with the aforementioned three proteins to assess their relative amount (23). 

When autophagy is moderately augmented, it exerts protective effects on cell function, such as ATP production and clearance of oxidized proteins and injured organelles. On the other hand, excessive activation of autophagy is associated with the risk of eliminating essential proteins and intracellular organelles, which can cause cell death (24). 

Chemical chaperones induce the CMA pathway, as the ability to mimic the function of chaperones in the autophagy pathway. They enhance the transfer of mutated and misfolded proteins and improve the folding capacity of the endoplasmic reticulum (25). The 4-phenyl butyric acid (4-PBA) is a non-toxic chemical chaperone and a type of fatty acid with low molecular weight. The American Food and Drug Administration has approved the application of 4-PBA (26) which is clinically used as an ammonia scavenger in children with urea cycle disorder and the treatment of sickle cell anemia and thalassemia (27). It has maximum uptake by the heart and kidneys and was recently manufactured in controlled release form due to its short half-life (28, 29). Previous *in vivo* studies have documented the potential efficacy of 4-PBA in reducing complications due to ischemia-reperfusion, fibrosis, and cardiac necrosis in rats (30, 31). There is a gap in existing knowledge in the area of the effects of chemical chaperones such as 4-PBA on MI. Thus, this *in vivo* study aimed to assess the role of 4-PBA on hemodynamic and histopathological factors following induction of MI by isoproterenol in the rats. In addition, proteins involved in autophagy such as Beclin1, LCII/I, and P62 are evaluated to further understand the compensatory mechanisms between the autophagic pathways in MI.

## Materials and Methods

***Chemical reagents***

Isoproterenol (Sigma Chemicals, USA), 4-PBA (Merck, Germany), Ketamin and (Alfasan, Netherland), Xylazine (Alfasan, Netherland), Acepromazine (Alfasan, Netherland), and Formaldehyde (Merck, Germany) were used in this study. The other reagents were of commercial analytical grade. 

***Experimental animals***

In this study, a total of 30 male Wistar rats weighing 270–300 g were selected and randomly assigned to six standard propylene cages (n=5 in each cage). The rats were housed at 25–27 °C and 50±10 % humidity under 12 hr light/12 hr dark cycles with *ad libitum* access to food and water. The technician taking care of the rats was blinded to the study objectives. In case of the appearance of a wound, infection, or death of a rat during the study period, it was excluded from the study and replaced with another rat.

This study was conducted under the institutional guidelines for the care and use of animal models. The approval by the ethics committee of Tabriz University of Medical Sciences was obtained (IR.TBZMED.VCR.REC.1397.139 and IR.TBZMED.VCR.REC.1397.048). In addition, the study was designed and implemented under the recommendations of the ARRIVE guidelines for reporting animal research (32). 

***Experimental procedures***

The rats were randomly divided into five groups (n=6): healthy control, isoproterenol, and three doses of 4-PBA (P20, P40, and P80) using a simple randomization software (Microsoft Excel 2016; Microsoft Co., Redmond, WA, USA).

The healthy control rats received a twice-subcutaneous injection of 0.3 ml saline at the back of their necks at a 24-hr interval. In addition, they received five injections of saline (0.3 ml, IP) at 24-hr intervals. In the isoproterenol group to induce MI, isoproterenol was dissolved in saline at a dosage of 100 mg/kg and subcutaneously injected into the back of the rat’s neck (in two consecutive days with a 24-hr interval). In addition, similar to the control group, saline (0.3 ml, IP) was injected daily for five days starting from the first day. 

The rats in groups P20, P40, and P80 received five intraperitoneal injections of 4-PBA at dosages of 20, 40, and 80 mg/kg, respectively. Simultaneously, all 4-PBA groups received two injections of isoproterenol, the same as the isoproterenol group. The surgical procedure was performed 96 hr after the second isoproterenol injection. NaOH was used for better dissolution of 4-PBA, and the pH was adjusted to 7.4 (33). The addition of NaOH converted the weak acid of 4-PBA, to sodium salt, similar to the saline group. Dose selection was based on previous similar cardiac studies of 4-PBA with the addition of lower doses to examine different dose ranges (30, 31).

On day 6 (96 hr after the second isoproterenol injection), the rats in all five groups were anesthetized by intraperitoneal injection of ketamine (40.5 mg/kg BW), xylazine (8.0 mg/kg BW), and acepromazine (0.4 mg/kg BW) and underwent a surgical procedure (34).

***Measurement of hemodynamic parameters***

An incision was made in the midline of the animal’s neck and the left carotid was isolated and a small incision was made in it to record heart rate (HR) and mean arterial pressure (MAP) by a catheter. Then, the open chest technique was used to assess the left ventricle function and direct measurement of left ventricle pressure alterations. For this purpose, the chest was opened and a catheter was directly inserted into the left ventricle. The pressure change at a fixed ventricular pressure (LV dP/dt/P), left ventricular maximal and minimal rates of pressure increase (LV dP/dt_min_ and LV dP/dt_max_), left ventricular systolic pressure (LVSP) and left ventricular end-diastolic pressure (LVEDP), mean arterial pressure (MAP), and heart rate (HR) were recorded (35, 36). 

***Blood collection and preparing the slides***

Once the abdomen of the rats was opened, the blood was collected from the hepatic portal vein using a 10-ml syringe. One drop of blood was placed on a slide and spread. After drying, it was fixed by dripping methanol over it and stained by Giemsa stain (Merck). The peripheral blood neutrophils were evaluated under a microscope (Olympus, Japan) at x100 magnification. The remaining blood was stored in the blood collecting tubes and plasma was separated from the blood cells by cold centrifugation (Eppendorf AG, Germany) at 3200 rpm for 20 min.

***Cardiac examination***

After opening the chest, the heart was removed and rinsed with cold saline at 4 °C and weighed. To assess cardiac hypertrophy and edema, the ratio of the heart weight (in grams) to the rat body weight (in kilograms) was calculated. The cardiac tissue of 15 rats was frozen at -70 °C for Western blotting and the others were fixed in 10% formalin for histopathological studies. Fixed tissues were embedded in paraffin and sectioned into pieces with 5-µm thickness. The specimens were stained with hematoxylin and eosin (Sigma Aldrich, USA) and inspected under a microscope at x40 magnification. Cardiac fibrosis and necrosis were assessed in each section by the morphometric point-counting procedure (37). A blinded technician scored the histopathological changes as mild (score 1), moderate (score 2), severe (score 3), or very severe (score 4) according to the degree of inflammation, myocardial disorganization, and myofibrillar loss. The myocardial pathologies were assessed according to the Association for European Cardiovascular Pathology guidelines (38).

***Measuring the serum TAC***

The serum TAC was measured in plasma samples and assessed using the Ferric reducing ability of plasma (FRAP) method (39). The method is based on the principle of the reduction of the ferric-tripyridyltriazine complex to the ferrous form by the anti-oxidants of a sample, which causes the development of an intense blue color that is spectrophotometrically measurable at 593 nm and the change in absorbance is related to the anti-oxidant capacity of the sample.

***Western blotting***

A total of 40 mg/kg of the cardiac tissue apex was dissected and lysed in 2 ml of the lysis buffer using a homogenizer. Then centrifuged at 1000 rpm, 4 °C for 10 min and the supernatant was stored at -70 °C for further analysis. For Western blotting, first, total protein was quantified by NanoDrop (Thermo, USA). Next, 50–100 µg protein was removed from the solution and electrophoretically transferred onto a polyvinylidene fluoride membrane using SDS-PAGE (12%). The membranes were blocked with 6% nonfat dried milk in phosphate-buffered saline (pH of 7.2) for one hour at room temperature. After blocking the non-specific binding sites, the membranes were rinsed with the wash buffer and placed in a solution containing primary antibodies and incubated overnight at 4 °C. The primary antibodies included the rabbit polyclonal anti-Beclin1 (1:1000; Cell Signaling, USA), rabbit polyclonal anti-P62 (1:1000; Cell Signaling, USA), rabbit polyclonal anti-LC3 (1:1000; Cell Signaling, USA), and mouse polyclonal anti-GAPDH (1:2500; Abcam, England). Next, all membranes were rinsed with the wash buffer and incubated at room temperature for one hour in a solution containing secondary antibodies such as goat anti-rabbit IgG horseradish peroxidase-conjugated secondary antibody (1:2000; Cell Signaling, USA) for Beclin1, P62, and LC3, and goat anti-mouse IgG horseradish peroxidase-conjugated secondary antibody (1:5000; Abcam, England) for GAPDH. The proteins were detected by the chemiluminescence method, and the appeared bands were observed by an imaging device. The optical density of the bands was quantified by image scanning analysis software (Image j; Wayne Rasband, National Institute of Health, USA) and reported as optical density per square millimeter.

***Statistical analysis***

Data were analyzed using SPSS version 25 (SPSS Inc., IL, USA). The data were presented as mean ± standard error of the mean (SEM). ANOVA was used to compare the data between the study groups. In case of the presence of a significant difference, Fisher’s least significant difference (LSD) test was applied. Paired t-test was performed for semiquantitative scoring data in pathology research. Differences between groups were considered significant if *P<*0.05.

## Results

***Hemodynamic parameters***

As demonstrated in Table 1, isoproterenol made a significant decrease in MAP and LVSP (*P*<0.01 and *P*<0.001, respectively versus the healthy control group). MAP in P20 and P40 groups were significantly higher (*P*<0.001, *P*<0.01, respectively) compared with isoproterenol. In the same way, 4-PBA significantly augmented LVSP in the P20 and P40 groups (*P*<0.001). 4-PBA in the concentration of 80 mg/kg could make no significant difference in MAP and LVSP. 

Heart rate decreased in the isoproterenol group (*P*<0.01). No significant change was documented in HR in groups P20, P40, and P80 compared with isoproterenol. After receiving isoproterenol, LVEDP increased by more than five times (*P*<0.001), which 4-PBA could diminish in P20, P40, and P80 groups (*P*<0.01, *P*<0.001, and *P*<0.01, respectively). 

The Isoproterenol group had lower LV dP/dt_max_ and LV dP/dt_min_ than the control group (*P*<0.01, *P*<0.05, respectively). P20 increased both the max and min LV dP/dt (*P*<0.01, *P*<0.05, respectively). P40 only made a significant increase in LV dP/dtmax (*P*<0.05). In contrast, max and min LV dP/dt were not effectively changed by P80 compared with isoproterenol. The results showed that the trend of the changes in LV dP/dt/P was similar to the changes in min LV dP/dt. 

***Effect of 4-PBA on the heart weight/body weight ratio***

**Figure 1:**
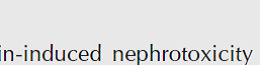
Effects of 4-Phenyl butyric acid (4-PBA) on heart weight/body weight ratio. The data were expressed as mean ± SEM (n=6). The ratio of the heart weight (HW, in grams) to the rat body weight (BW, in kilograms) was calculated. Iso: isoproterenol; P20: 4-PBA (20 mg/kg) + isoproterenol; P40: 4-PBA (40 mg/kg) + isoproterenol; P80: 4-PBA (80 mg/kg) + isoproterenol. **P<0.01 from the control group using ANOVA and LSD *post hoc* test

To assess cardiac hypertrophy and edema, the ratio of the heart weight (in grams) to the rat body weight (in kilograms) was calculated and demonstrated in Figure 1. The heart weight/body weight ratio increased in the isoproterenol group (*P*<0.01). 4-PBA in none of the treated groups could remarkably influence this ratio compared with isoproterenol (*P*>0.05).

***Effect of 4-PBA on cardiac tissue histopathology***

**Figure 2:**
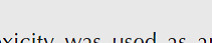
Photomicrographs of sections of rat cardiac apexes for evaluating necrosis, tissue disorientation, and neutrophil accumulation. The rat cardiac tissue in the isoproterenol group showed fibrosis, cardiomyocyte necrosis, and local infiltration of mononuclear cells (black arrows). Treatment with 40 mg/kg 4-Phenyl butyric acid caused a significant improvement. Sectioned at five micrometer and stained with Hematoxylin and Eosin (H&E) (40M). Quantitative analysis was performed by grading histopathological changes. Results presented as mean ± SEM (n = 5). Iso: isoproterenol; P20: 4-PBA (20 mg/kg) + isoproterenol; P40: 4-PBA (40 mg/kg) + isoproterenol; P80: 4-PBA (80 mg/kg) + isoproterenol. ***P<0.01 from control group, ###*P*<0.001 from Iso group using Kruskal-Wallis test

As shown in Figure 2, in the control group, myocardial fibers were arranged regularly with distinctive striation. No clear degeneration or necrosis was noted. Histological sections of cardiac tissue in the isoproterenol group showed extensive subendocardial necrosis, hypertrophy, abundant fibroblastic hyperplasia, capillary dilation, and leukocyte infiltration. P40 with less amount of these isoproterenol-induced injuries significantly decreased the inflammatory response and destruction (*P*<0.001). In contrast, changes in groups P20 and P80 were not significant (*P*>0.05). To report the quantitative analysis of histological results, grading of histopathological changes of heart tissue after isoproterenol injection and in the presence of different doses of 4-PBA are also shown in Figure 2.

***Effect of 4-PBA on the peripheral blood neutrophils and serum TAC***

**Figure 3:**
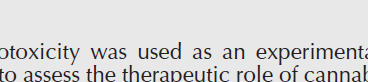
Effects of 4-Phenyl butyric acid (4-PBA) on the percentage of peripheral blood neutrophils. The data were expressed as mean ± SEM (n=6). Iso: isoproterenol; P20: 4-PBA (20 mg/kg) + isoproterenol; P40: 4-PBA (40 mg/kg) + isoproterenol; P80: 4-PBA (80 mg/kg) + isoproterenol. ***P<0.01 from the control group, ##*P*<0.01, ###*P*<0.001 from the Iso group using ANOVA and LSD *post hoc* test

The peripheral blood neutrophils count and serum TAC are demonstrated in Figure 3. Following the injection of isoproterenol and induction of inflammation, the percentage of peripheral blood neutrophils in the isoproterenol group was significantly augmented compared with the control group (*P*<0.001). This ratio decreased in all 4-PBA receiving groups with a significant difference in P40 (*P*<0.01) and P80 (*P*<0.001). 

The serum TAC significantly decreased in the isoproterenol group compared with the control group (*P*<0.001). Using the 80 mg/kg of 4-PBA, made a notable serum TAC augmentation (*P*<0.001 *versus *the isoproterenol group). P20 and P40 had no considerable TAC variation (*P*>0.05).

***Results of Western blotting***

**Figure 4.**
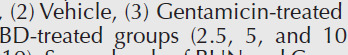
Effect of 4-Phenyl butyric acid (4-PBA) on Beclin1/GAPDH ratio. The data were expressed as mean ± SEM (n=4). Iso: isoproterenol; P20: 4-PBA (20 mg/kg) + isoproterenol; P40: 4-PBA (40 mg/kg) + isoproterenol; P80: 4-PBA (80 mg/kg) + isoproterenol. The samples derive from the same experiment or parallel experiments and the gels/blots were processed in parallel

The Beclin1/GAPDH protein ratio increased in the isoproterenol group compared with the control. An insignificant change in P20, P40, and P80 groups was documented for Beclin1/GAPDH compared with the isoproterenol group (*P*>0.05, Figure 4). 

**Figure 5:**
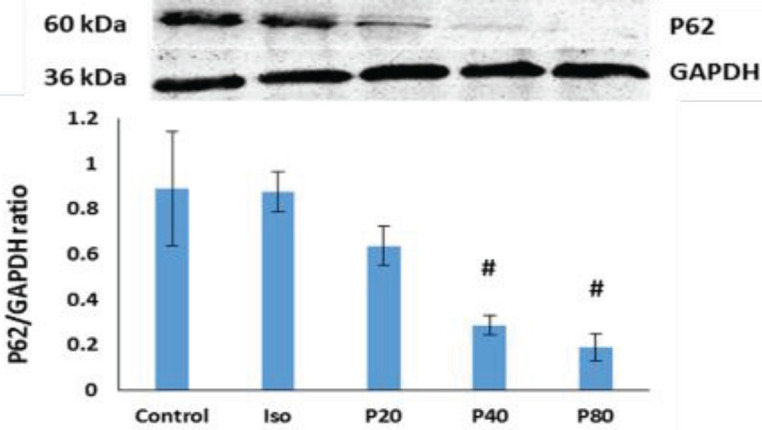
Effect of 4-Phenyl butyric acid (4-PBA) on P62/GAPDH ratio. The data were expressed as mean ± SEM (n=4). Iso: isoproterenol; P20: 4-PBA (20 mg/kg) + isoproterenol; P40: 4-PBA (40 mg/kg) + isoproterenol; P80: 4-PBA (80 mg/kg) + isoproterenol. #P<0.05 from isoproterenol treated group using ANOVA and LSD *post hoc* test. The samples derive from the same experiment or parallel experiments and the gels/blots were processed in parallel

The difference in P62/GAPDH protein level between the isoproterenol and the healthy control groups was not remarkable (*P*>0.05). However, P40 and P80 significantly decreased the P62 expression compared with the isoproterenol group (*P*<0.05, Figure 5). 

**Figure 6:**
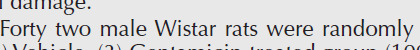
Effect of 4-Phenyl butyric acid (4-PBA) on LC3-II/LC3-I/GAPDH ratio. The data were expressed as mean ± SEM (n=4). Iso: isoproterenol; P20: 4-PBA (20 mg/kg) + isoproterenol; P40: 4-PBA (40 mg/kg) + isoproterenol; P80: 4-PBA (80 mg/kg) + isoproterenol. The samples derive from the same experiment or parallel experiments and the gels/blots were processed in parallel

The ratio of LC3-II/LC3-I/GAPDH protein was insignificantly higher in the isoproterenol group compared with the control (*P*>0.05). In groups receiving 4-PBA, this amount decreased in the P20 group and again increased in the P40 and P80 groups compared with the isoproterenol group, however, none of these changes were statistically significant (*P*>0.05, Figure 6). 

## Discussion

This study assessed the cardiac pharmacological effects of 4-PBA in rats with isoproterenol-induced MI. The hypothesis was that injection of 4-PBA by interfering in autophagy pathways would improve the pathological status of the heart. The results showed that cardiac injury caused by isoproterenol significantly decreased following the administration of 4-PBA. 

Isoproterenol is a beta-agonist sympathetic stimulant that induces MI, changes the hemodynamic parameters, and causes necrosis, fibrosis, inflammation, and neutrophil accumulation (40). An increase in incompetent protein levels occurs in oxidative stress and MI, which results in further damage (41). The cardiac status after MI may be improved by the potential of 4-PBA in the regulation of oxidative stress and elimination of unfolded and misfolded proteins (42).

In this study, administration of 4-PBA in dosages of 20 and 40 mg/kg significantly improved the hemodynamic parameters. They reversed all the heart-weakening effects of isoproterenol by increasing MAP, LV dP/dt/P, LV dP/dt_max_, LV dP/dt_min_, and LVSP and decreasing LVEDP. Considering the LV dp/dt/P results, the mechanism of 4-PBA to demonstrate these hemodynamic influences is highly by enhancing the cardiac muscle power and less likely through changing the volume or preload. In contrast, the variation in hemodynamic parameters was not significant following the injection of 80 mg/kg of 4-PBA. This controversy can be due to excessive stimulation of autophagy by the higher dose of 4-PBA and the consequent elimination of essential proteins of cells, which could induce apoptosis in cardiac cells (43, 44). 

Administration of 4-PBA in the concentration of 40 mg/kg decreased necrosis, fibrosis, and inflammation caused by isoproterenol in cardiac tissue. Previous research on the effect of 4-PBA on cardiac necrosis and fibrosis indicated that injection of 80 mg/kg 4-PBA 1 hr before induction of MI decreased fibrosis and necrosis in rats by minimizing oxidative stress (31). Another study assessed the efficacy of 4-PBA to prevent the effects of thapsigargin on cardiac fibroblasts isolated from rats. It showed that 4-PBA decreased the endoplasmic reticulum stress and accumulation of procollagen in cardiac fibroblasts and consequently decreased cell death (45). Our results were in line with their findings with the difference that 40 mg/kg of 4-PBA in our study corresponded to approximately 80 mg/kg dosage of 4-PBA sodium salt in previous studies. 

Administration of 40 and 80 mg/kg of 4-PBA had a striking effect in reducing the percentage of peripheral blood neutrophils, while this fluctuation was not significant following the injection of 20 mg/kg of 4-PBA. This finding can be due to the effective anti-inflammatory role in higher doses of 4-PBA, which have been previously reported. A study was performed to assess the efficacy of 4-PBA in palliation of inflammation caused by dextran sulfate sodium (DSS) in mice colons. It exerted anti-inflammatory effects at the higher dose of 150 mg/kg by suppressing the nuclear factorκB activation and inhibiting the pro-inflammatory cytokines, interleukin-6, interleukin-1B, and tumor necrosis factor-alpha (46). 

Compatible with neutrophil count abatement, the increase in serum TAC was only significant in a high dose of 80 mg/kg of 4-PBA. This result can be related to the decline of oxidative stress, which results in increasing the anti-oxidant capacity and decreasing cell damage (47). 

The ratio of heart weight/body weight significantly increased following the injection of isoproterenol compared with the control group. This increase seems to be due to edema and cardiac tissue inflammation and is less likely related to cardiac tissue hypertrophy; as we assessed acute MI and there was not sufficient time for cardiac hypertrophy occurrence. Injection of 4-PBA did not cause a significant change in this ratio, which indicates the inefficacy of 4-PBA for tissue edema. 

The results of Western-blotting tests showed that the level of P62 significantly decreased following 40 and 80 mg/kg of 4-PBA, indicating the induction of the macro-autophagy pathway. In addition, the variation trend of other proteins in this cascade was also towards the induction of macro-autophagy, though not significant. There are different reports introducing the 4-PBA as a chemical chaperon with the potential of inducing the CMA pathway. Considering the correlation of CMA with the macro-autophagy pathways, by induction of CMA, the macro-autophagy pathway is suppressed (48-50). In this study, in contrast to our expectations, injection of 4-PBA induced the macro-autophagy pathway. This may be due to a sudden increase in unfolded or misfolded proteins in MI, which can activate both macro-autophagy and CMA pathways at the same time (51). Moreover, P62 can serve as a mediator between the CMA and macro-autophagy pathways, suggesting the cross-talking of these two pathways (52). Obtaining sinus rhythm in effective results of different dosages of 4-PBA can show the fact that altering the autophagy and CMA are so dose-dependent and can occur in a special optimum concentration of 4-PBA.

Furthermore, differences in effective doses can be due to differences in the targets of this medication. Tissue and hemodynamic changes probably show different patterns in response to the administration of 4-PBA. In general, according to the obtained results and the bilateral activity of autophagy, it seems that the optimum dose of 40 mg/kg in this study, is more effective in improving the hemodynamic, anti-inflammatory, and histological effects. Future studies with a deep focus on CMA pathways and markers are required on the cardiac functions of 4-PBA.

**Table 1:** Effects of 4-Phenyl butyric acid (4-PBA) on hemodynamic parameters and left ventricular function of rat

**Groups **	**MAP (mmHg)**	**HR** **(bpm)**	**LVSP (mmHg)**	**LVEDP (mmHg)**	**LV dP/dt/P (1/sec)**	**LV dP/dt**_max_ ** (mmHg/sec)**	**LV dP/dt**_min_ **(mmHg/sec)**
**Control**	74±8.5	275.1±5.7	71.6±3.7	2.6±0.8	4.9±0.6	1146±91	-964±168
**Iso**	49.2±6.2^**^	217±6.3^**^	35.6±2.9^***^	12.6±1.5^***^	1.2±0.3^***^	582± 117^ **^	-546±159^*^
**P20**	87.2±4.8^###^	217.5±8.6	77±1.9^###^	5.4±1^ ##^	4±0.4^##^	1146±106^##^	-1039±121^#^
**P40**	76.1±3.9^##^	235.6±10.5	79.5±5.9^###^	4.6±0.8^###^	3.1±0.4^#^	952.5±131^#^	-835±165
**P80**	46.2±4.5	212.24±25.1	43.67±3	7.4±1.4^##^	1.8±0.7	440±52	-356.5±49

Data are expressed as mean ± SEM (N=6). MAP: mean arterial pressure; HR: heart rate; LVSP: left ventricular systolic pressure; LVEDP: left ventricular end diastolic pressure; LV dp/dt/P: lower rate of pressure change at a fixed ventricular pressure; LV dP/dtmax and LVdP/dtmin: Left ventricular maximal and minimal rates of pressure increase. Iso: isoproterenol; P20: 4-PBA (20 mg/kg) + isoproterenol; P40: 4-PBA (40 mg/kg) + isoproterenol; P80: 4-PBA (80 mg/kg) + isoproterenol. **P<0.01, ***P<0.001 from the control group; #*P*<0.05, ##*P*<0.01, ###*P*<0.001 from Iso group using one-way ANOVA with LSD *post hoc* test

## Conclusion

The current results showed that injection of 4-PBA caused significant improvements in hemodynamic factors, histopathological changes, inflammation, serum TAC, and neutrophil accumulation, which demonstrated the cardio-protective effects in isoproterenol-induced MI. As the autophagy modulation potential of 4-PBA, understanding its compensatory mechanisms between the different autophagic pathways is important in light of the development of interventions aimed at therapeutic purposes with altering autophagy, especially in cardiac disorders. 

## Authors’ Contributions

FV provided project administration and wrote the original draft; AG supervised; HV helped with writing, review, editing, and funding acquisition.

## Conflicts of Interest

The authors declare that they have no conflicts of interest.
